# Biofilm dynamics: linking in situ biofilm biomass and metabolic activity measurements in real-time under continuous flow conditions

**DOI:** 10.1038/s41522-020-00153-9

**Published:** 2020-10-21

**Authors:** Kyle B. Klopper, Riaan N. de Witt, Elanna Bester, Leon M. T. Dicks, Gideon M. Wolfaardt

**Affiliations:** 1 Department of Microbiology, Stellenbosch University, Stellenbosch, South Africa; 2 Department of Chemistry and Biology, Ryerson University, Toronto, ON Canada

**Keywords:** Biofilms, Water microbiology

## Abstract

The tools used to study biofilms generally involve either destructive, end-point analyses or periodic measurements. The advent of the internet of things (IoT) era allows circumvention of these limitations. Here we introduce and detail the development of the BioSpec; a modular, nondestructive, real-time monitoring system, which accurately and reliably track changes in biofilm biomass over time. The performance of the system was validated using a commercial spectrophotometer and produced comparable results for variations in planktonic and sessile biomass. BioSpec was combined with the previously developed carbon dioxide evolution measurement system (CEMS) to allow simultaneous measurement of biofilm biomass and metabolic activity and revealed a differential response of these interrelated parameters to changing environmental conditions. The application of this system can facilitate a greater understanding of biofilm mass–function relationships and aid in the development of biofilm control strategies.

## Introduction

The field of microbial ecology has readily embraced the importance of biofilms as the predominant mode of growth, juxtaposed to the broader scientific community that has only paid closer attention to this mode of microbial proliferation in the last two decades^1,2^. Bacteria rarely exist and proliferate as individual cells, but rather form complex community structures such as flocs and biofilms^3^. Biofilms are complex and highly intra- and interlinked aggregates of cells (single species or mixed species) housed within an equally multifaceted and robust extracellular matrix^4^. This preferential sessile growth mode ensures that microbial communities are ubiquitous throughout the natural and man-made environment^5–7^. The ability to colonize and persist in diverse environments can be mutually beneficial to mankind (bioremediation, waste processing etc.), however it can be equally destructive and deleterious (biofouling, nosocomial infections etc.) in action^3,6,7^. Biofouling of submerged surfaces result in detrimental and/or destructive consequences to surfaces or processes, costing billions of dollars to manage^6,8^. Besides the financial implications associated with biofouling, biofilm formation imposes a serious burden to human health from a medical or food safety point of view. Biofilm-associated contamination of food, pharmaceutical products or medical equipment is well-documented^6,9^. Furthermore, current estimates show that between 65 and 80% of all infections are associated with biofilms^10–13^.

Even though biofilm studies date back by more than a century, it is only in the last 50 years that the scientific community started developing tools for the in-depth study of microbial biofilms^7,14,15^. Molecular based approaches are the predominant method in which biofilm biology is studied^16–18^. The vast majority of existing tools have been modified from other fields of study and tend to be destructive in nature resulting in an inadvertent end-point analysis^14^. In addition, the complexities inherent to some techniques (microscopy, flow cytometry etc.) result in low resolution/frequency of biofilm analysis due to the time constraints of these techniques^19^.

In addition, the toolbox of available analytical techniques represents methods that have their origin in planktonic sampling that cannot necessarily be applied to sessile populations. For example, aggregate formation, limited biomass, extracellular polymeric substance (EPS) production, or heterogeneous growth rates inherent to biofilms should also be considered. Disregarding these factors can limit the information garnered from such techniques, especially as it is often applied at fixed intervals. Furthermore, cultivation of biofilms in batch systems results in simultaneous nutrient depletion and metabolite accumulation without external replenishment and removal, respectively, which do not realistically simulate open systems where biofilms modulate their response to changing environmental conditions to maintain some degree of equilibrium. Batch cultivation may thus provide a biased view, which does not take the stochastic nature of biofilms into account. Specialized techniques, including confocal laser scanning microscopy and attenuated total reflectance Fourier-transform infrared spectroscopy have been developed to analyze biofilm biomass with a high degree of success. Unfortunately, these techniques are often complex, require significant optimization as well as large, intricate and expensive pieces of equipment^14,19^.

The broad uptake of “do-it-yourself” (DIY) electronics into advanced and complex scientific equipment, enabled by the “internet of things” (IoT), component availability and easy to deploy programming interfaces have allowed for the expansion of electronics into other fields^20^^,^^21^ and facilitated the trickle-down of technology previously affordable primarily by high-tech companies and research organizations (https://openpcr.org/)^20,22–24^. This greater access facilitated the application of IoT sensors and tools to answer biological questions and the creation of a new subfield known as DIY biology^20,22^.

Here we report on the BioSpec system, which we have developed to study microbial biofilms. The BioSpec is an affordable, modular, nondestructive, and real-time monitoring system that detects changes in biomass formation in situ and under continuous flow. Combined with the carbon dioxide evolution measurement system (CEMS)^15^, this approach allows accurate real-time monitoring of changes in biofilm biomass and metabolism, in response to environmental fluctuations.

## Results

### Validation of BioSpec: turbidity assay

**Fig. 1:**
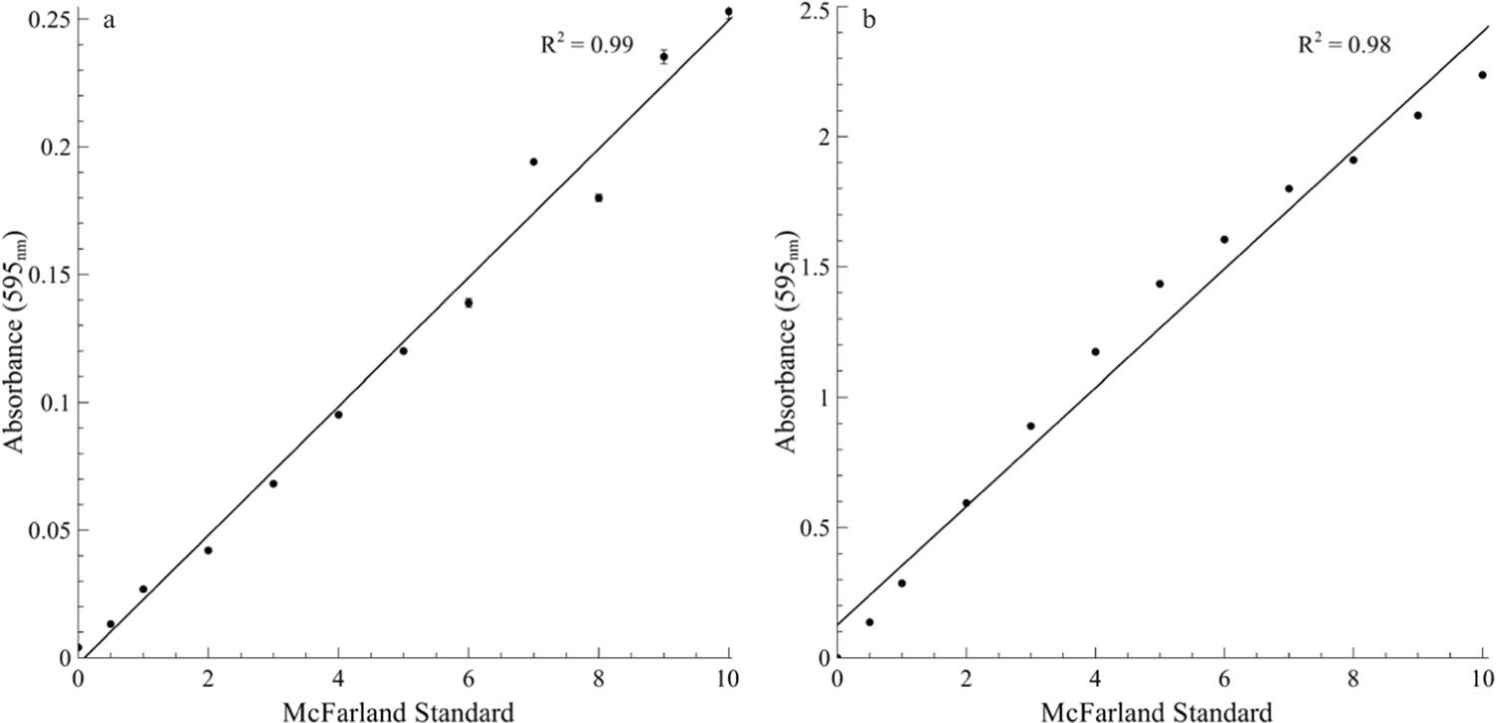
Comparative absorbance measurements at 595 nm for a range of McFarland turbidity standards. The change in absorbance values was determined for an increasing range of McFarland turbidity standards with the BioSpec system (**a**) and a conventional spectrophotometer (**b**). An increase in turbidity results in a decrease in transmission of light coupled with an increase in absorbance. Simple linear regression was performed and the corresponding *R*^2^ values are indicated on each graph (*p* < 0.05). Each data point represents the average of three undiluted sample readings and error bars indicate the standard deviation (*n* = 3).

Changes in absorbance at 595 nm associated with increasing McFarland standards exhibited a linear correlation for BioSpec (*R*^2^ = 0.99, *p* < 0.05) (Fig. 1a) and spectrophotometer (Spectroquant^®^) (*R*^2^ = 0.98, *p* < 0.05) (Fig. 1b), respectively. Minimal variations were observed between replicates, as evident from small standard deviations (Fig. 1a, b).

### Planktonic growth determined with BioSpec

**Fig. 2:**
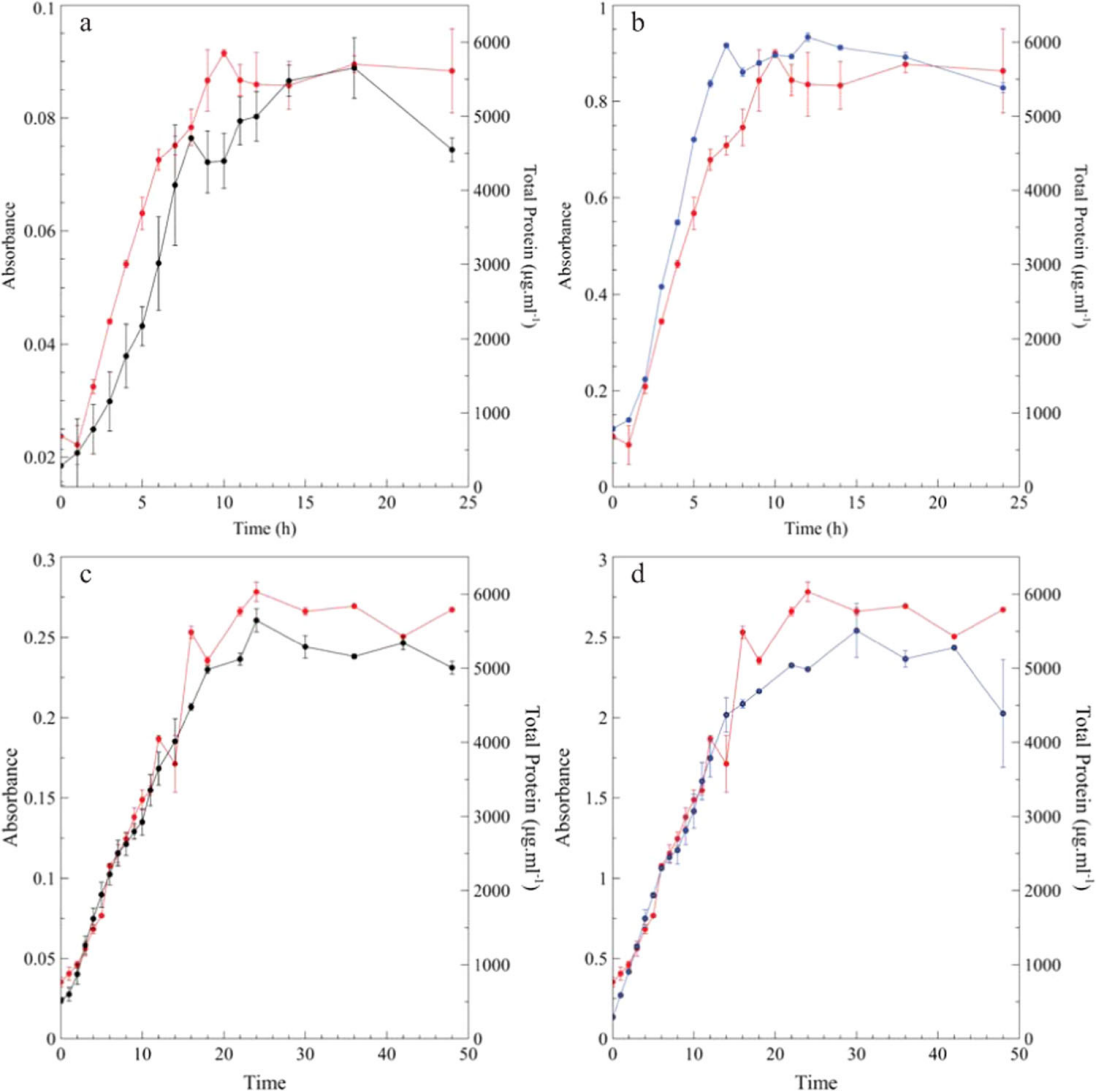
Planktonic *Pseudomonas aeruginosa* PAO1 growth curve. The growth curve of PAO1 cultivated aerobically in 3 g L^−1^ TSB (**a**, **b**) and 10 g L^−1^ TSB (**c**, **d**) at 26 °C for 24 to 48 h, respectively. Samples were taken at fixed intervals from the batch flasks, with biomass being measured optically with either the BioSpec (**a**, **c**, black lines) or Spectroquant™ (**b**, **d**, blue lines) and total protein analysis as an independent measure of biomass (red lines). Each data point represents the average of two independent flasks (biological duplicates) and sample triplicates with the error bar indicating the standard deviation (*n* = 6).

**Fig. 3:**
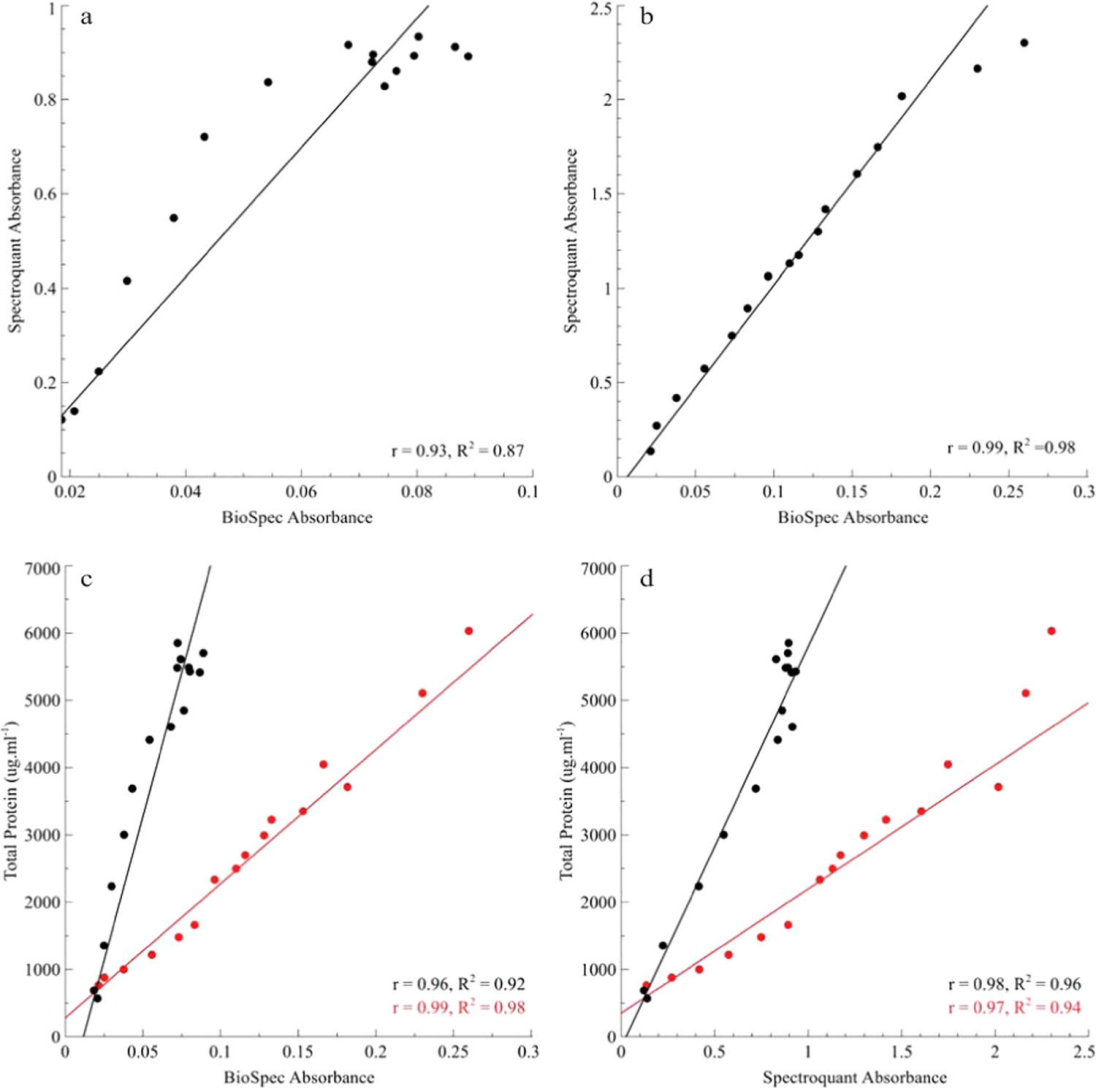
Direct correlations between BioSpec and Spectroquant absorbance measurements and total protein content of planktonic *P. aeruginosa* PAO1 biomass cultivated for 24–48 h in 3 and 10 g l^−1^ TSB. **a** Correlation between absorbance measurements from both BioSpec and Spectroquant for the biomass generated in 3 g l^−1^ TSB (*r* = 0.93, *R*^2^ = 0.87, *p* < 0.05). **b** Correlation between absorbance measurements from both BioSpec and Spectroquant for biomass generated in 10 g L^−1^ TSB (*r* = 0.99, *R*^2^ = 0.98, *p* < 0.05). **c** Correlation between total protein and BioSpec measurements for biomass derived from 3 g L^−1^ TSB (Black dots and line, *r* = 0.96, *R*^2^ = 0.92) and 10 g L^−1^ TSB (Red dots and line, *r* = 0.99, *R*^2^ = 0.98) respectively. **d** Correlation between total protein and Spectroquant measurements for biomass derived from 3 g L^−1^ TSB (Black dots and line, *r* = 0.98, *R*^2^ = 0.96) and 10 g L^−1^ TSB (red dots and line, *r* = 0.97, *R*^2^ = 0.94) respectively. Simple linear correlation was performed with the Pearson correlation coefficient (*r*) and coefficient of determination (*R*^2^) calculation for each correlation.

During planktonic cultivation of *P. aeruginosa* PAO1 in batch flasks containing 3 g L^−1^ Tryptic soy broth (TSB), the BioSpec accurately tracked the increase in planktonic biomass, following a similar sigmoidal curve to that of the total protein isolated from the biomass (Fig. 2a). Distinct log and stationary growth phases were recorded for total planktonic biomass and protein with both the BioSpec and Spectroquant^®^ (Fig. 2a, b). Similar trends were observed, albeit at higher absorbance values, when the nutrient concentration was increased ±3-fold to 10 g L^−1^ TSB (Fig. 2c, d). The higher nutrient concentration allowed for an increase in cell density and larger absorbance values, corresponding to the same range as that measured for McFarland standards 5–10 (Fig. 1a, b). Direct comparisons of absorbance measurements using the commercial spectrophotometer and BioSpec obtained from both the 3 and 10 g L^−1^ TSB planktonic growth experiments were performed (Fig. 3). Calculated linear correlations coefficients of *r* = 0.93 (3 g L^−1^ TSB, Fig. 3a) and *r* = 0.99 (10 g L^−1^ TSB, Fig. 3b) were observed, with the poorer correlation for the low nutrient concentration attributable to close clustering of the latter time points due to the onset of the stationary phase of growth (after 10 h of incubation, Fig. 2a). Strong linear correlations (*r* > 0.96) between total protein and absorbance values for both the BioSpec and conventional spectrophotometer were obtained for the planktonic biomass (Fig. 3c, d). Overall, BioSpec tracked changes in planktonic populations comparatively well over time when compared with a conventional spectrophotometer.

### Sessile growth determined with BioSpec

**Fig. 4:**
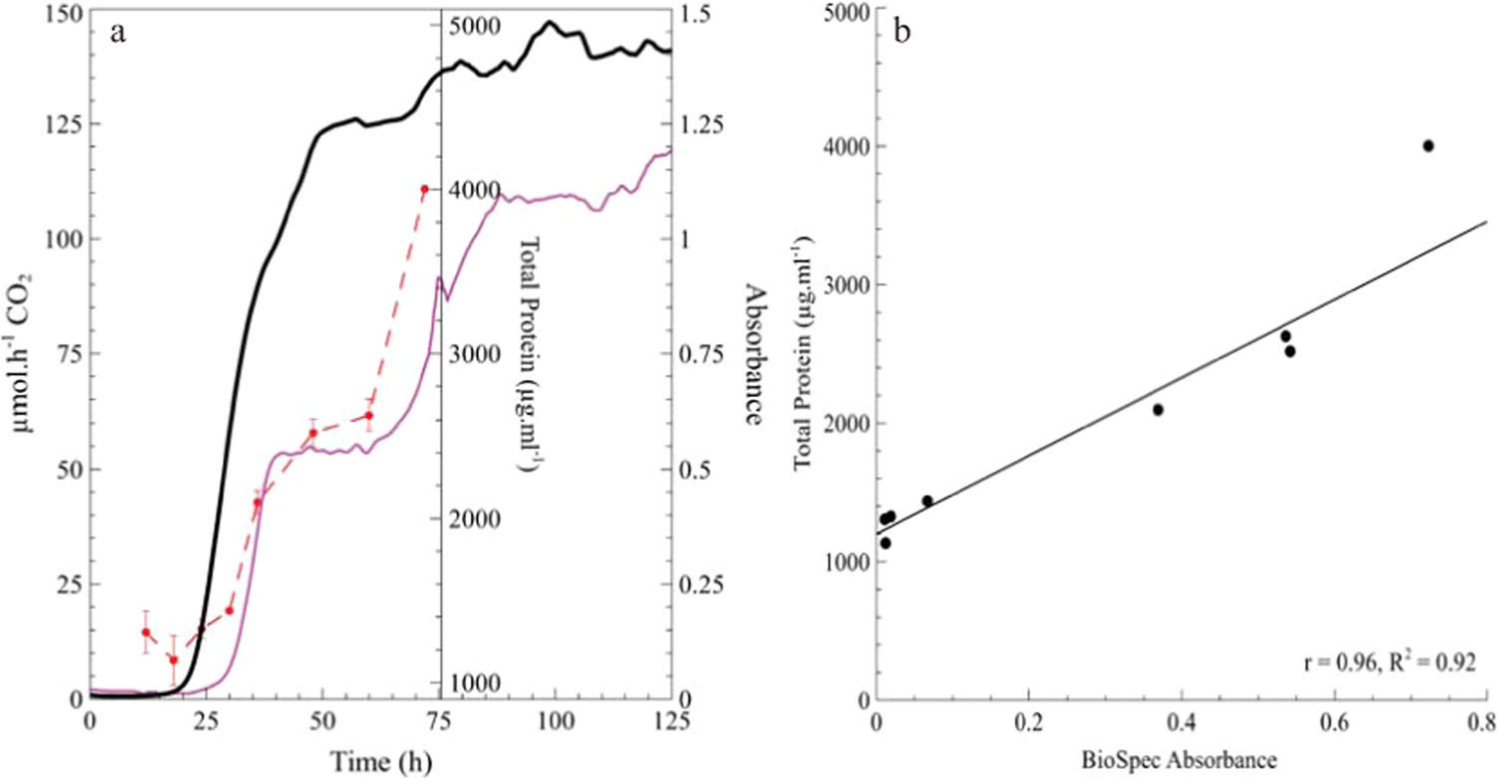
Sessile growth of *P. aeruginosa* PAO1 under continuous flow conditions. **a** PA01 biofilms were cultivated in the combined CEMS-BioSpec system with 10 g L^−1^ TSB supplied at a flow rate of 12.5 mL h^−1^. Changes in biofilm metabolic activity (black line) and biofilm biomass (magenta line) were monitored in real-time over the course of 125 h. Total protein from biofilm biomass (red dashed line) was sampled eight times during the first 75 h, each point is the mean of biological duplicates, technical triplicates with the standard deviation indicated by the error bar (*n* = 6). **b** Direct correlation of total protein isolated from the sessile biomass with the corresponding BioSpec absorbance measurements of attached biomass (*r* = 0.96, *r*^2^ = 0.92, *p* < 0.05). Simple linear correlation was performed with Pearson correlation coefficient (*r*) and coefficient of determination (*R*^2^) calculated for each correlation.

Biofilm development was monitored in real-time with the BioSpec and CEMS. Increases in total biofilm protein, indicative of an increase in biofilm biomass, corresponded to changes detected by the BioSpec (Fig. 4a). An increase in whole-biofilm metabolic activity by CEMS was detected within the first 15 h after inoculation (Fig. 4a). When comparing whole-biofilm metabolic activity (μmol h^−1^ CO_2_) to total biofilm protein (μg total protein mL^−1^) and biofilm biomass (absorbance at 595 nm), it is evident that the detection limit of metabolic activity is lower than that of the biomass measurements. After an exponential increase in the rate of CO_2_ production by cells in the biofilm, the respiration rate slowed down after ~50 h. The gradual increase in metabolic rate from 120 μm h^−1^ CO_2_ (±48 h, end of exponential phase) to 140 μmol h^−1^ CO_2_ (±80 h) and the maintenance of this rate until the end of experiment (125 h) is due to the presumed onset of steady state. Although a similar sigmoidal growth trend was observed for all biofilm metrics, both the total protein and BioSpec measurements indicate that substantial biofilm biomass was still being formed after 50 h of incubation, with a near doubling in the protein concentration and absorbance measurements between 50 and 70 h. Direct comparison of the total protein isolated from the biofilm and BioSpec absorbance measurements yielded a strong positive correlation (Fig. 4b, *r* = 0.96), thus further strengthening the utility of the BioSpec to accurately and nondestructively monitor changes in biofilm biomass over time. The combined application of BioSpec and CEMS not only facilitated the simultaneous measurement of two distinct but related biofilm metrics, but crucially allowed elucidation of the time-dependent dynamic variation in the different metrics.

### Simultaneous monitoring of nutrient availability on biofilms

**Fig. 5:**
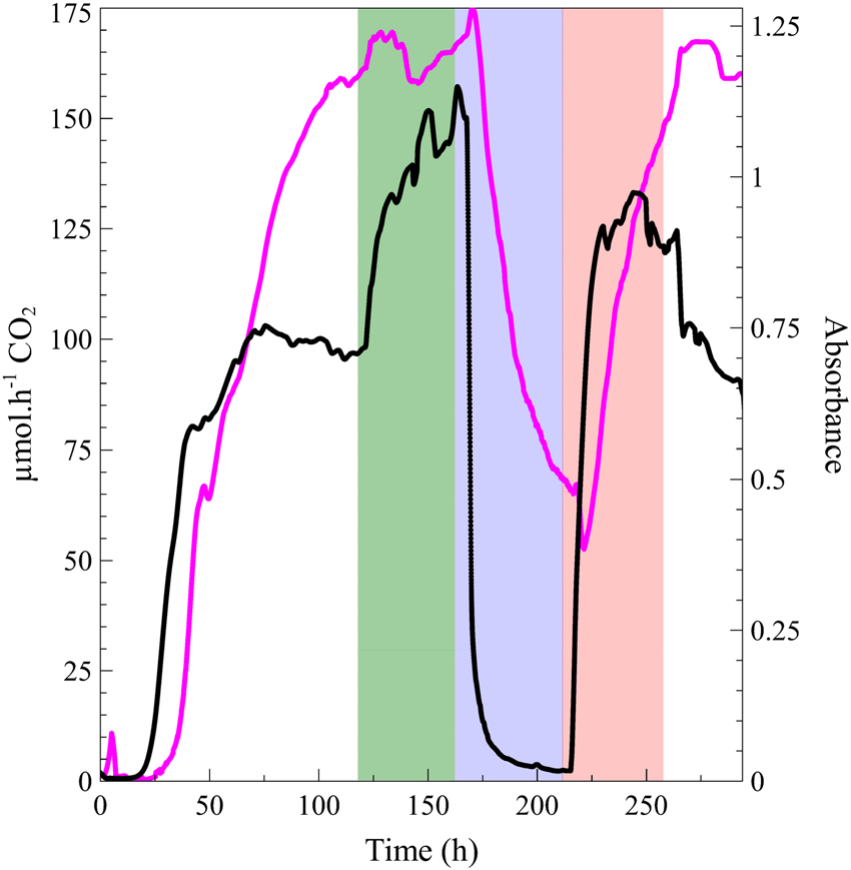
The effects of changes in nutrient availability on biofilm parameters. A *P. aeruginosa* PAO1 biofilm was initially cultivated under flow with a nutrient concentration of 3 g L^−1^ TSB until a steady state was reached in metabolic activity (black line) and approached in the case of biomass (magenta line) (0–122, left white region **h**). A threefold increase in nutrient concentration (10 g L^−1^ TSB) was introduced and the biofilm was again allowed to stabilize to a new steady state (122–164 h, dark green region) before the nutrient concentration was reduced tenfold (TSB buffer components only, 164–214 h, blue region). A new metabolic steady state was reached at ±215 h. After exposure of biofilms to this nutrient limited condition, the starvation phase was alleviated by the increase of nutrients to 6 g L^−1^ TSB and allowed to reach a metabolic steady state (214–264 h, red region). The final phase of treatment was to return the biofilm to the nutrient conditions on which the biofilm was established, 3 g L^−1^ TSB (264–300 h, right white region).

The *P. aeruginosa* PAO1 biofilm initially cultivated on 3 g L^−1^ TSB reached a steady state with respect to metabolic activity of ~100 μmol h^−1^ CO_2_ after 75 h (Fig. 5). In contrast, biomass continued to increase for another ±48 h prior to stabilizing at 120 h, with absorbance values of ~1.15. The introduction of 10 g L^−1^ TSB caused a rapid increase in both biofilm biomass and metabolic activity as more nutrients became available (122–164 h, dark green region, Fig. 5). However, while biofilm biomass increased by 10% (from absorbance of 1.15–1.23), a more pronounced increase in metabolic activity from 100 to 150 μmol h^−1^ (50% increase) was observed.

The most substantial change in all measured parameters can be seen from 164 to 214 h, where TSB containing only buffer components (no added carbon or nitrogen) was introduced into the system (blue region, Fig. 5). The lack of nutrients caused a rapid response in metabolic activity, with a 90% reduction in the CO_2_ production rate. This decrease was coupled to a slightly delayed, but substantial decline (60%) in attached biofilm biomass. The reintroduction of nutrients (6 g L^−1^ TSB from 214 to 264 h, red region, Fig. 5) resulted in a prompt response from both biofilm metrics. The biofilm biomass reestablished rapidly and while it did not reach a steady state during this interval, it arguably resulted in an increase in both the number of cells contributing to CO_2_ production as well as the cell-specific respiration rate. A steady-state production rate of 125 μmol h^−1^ CO_2_ was achieved within this period, which is approximately midway between that maintained on 10 g L^−1^ TSB (150 μmol h^−1^ CO_2_) and that of 3 g L^−1^ TSB (100 μmol h^−1^ CO_2_). Interestingly, the reintroduction of 3 g L^−1^ TSB (270 h, white region on right, Fig. 5) resulted in biofilm biomass similar to pre-perturbation steady state, while metabolic activity decreased by 25%.

### Simultaneous monitoring of chemical oxidation/disruption on biofilms

**Fig. 6:**
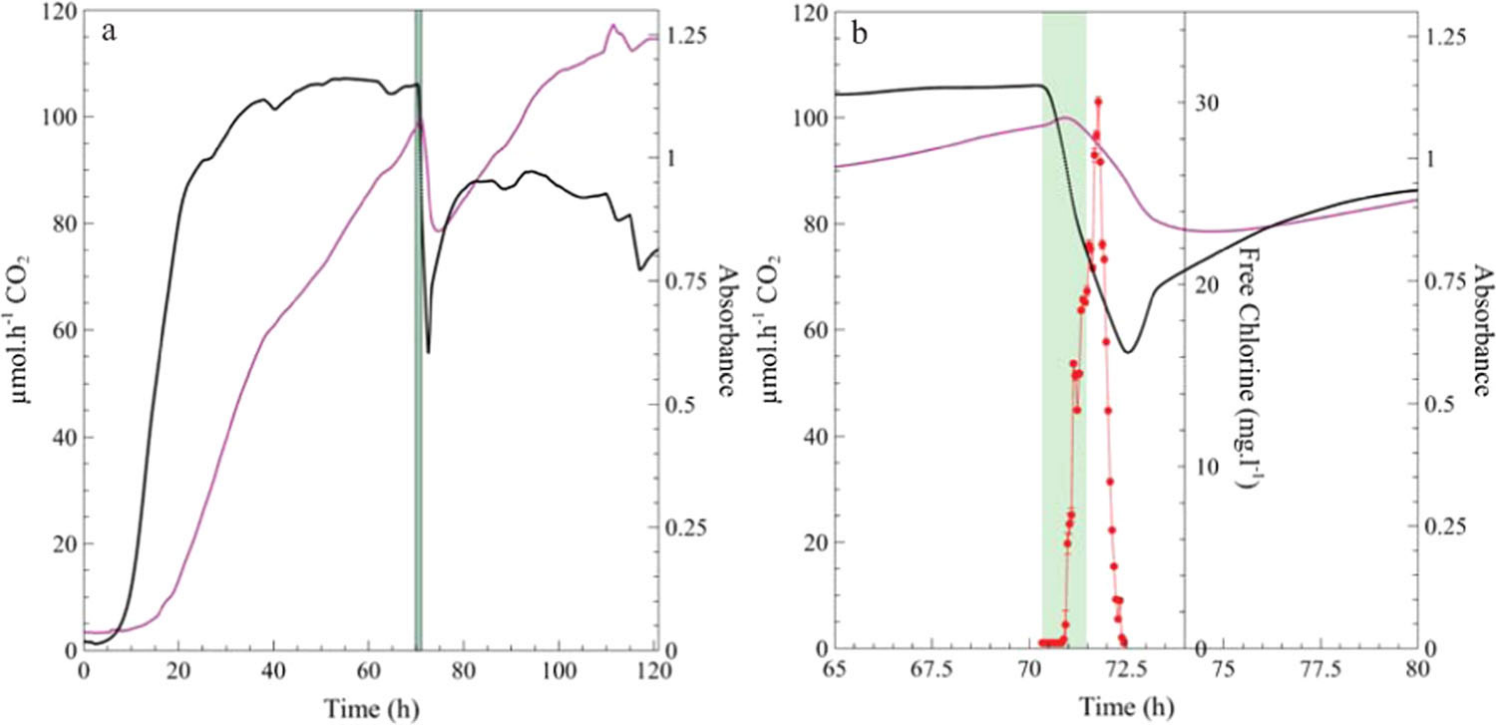
Chemical oxidation and/or disruption of the biofilm using a diluted commercial sodium hypochlorite solution. *P. aeruginosa* PAO1 biofilms were cultivated under flow with a nutrient concentration of 3 g L^−1^ TSB until whole-biofilm metabolic activity (black line) and approximate biomass (magenta line) steady-state was reached (0 ± 70 h) (**a**). The inflow of nutrient solution was replaced with a solution of diluted sodium hypochlorite diluted with dH_2_O (1:100, 570 ppm free chlorine). The sodium hypochlorite solution was aseptically introduced into the system using the same flow rate for a treatment period of 1 h (green shaded region) followed by reintroduction of nutrient medium (recovery phase, post 71 h). **b** Zoomed-in view showing a 15 h period spanning pre-perturbation and recovery phase. The free chlorine concentrations in the effluent from the CEMS-BioSpec system (red line) was determine prior to the start of the treatment period, during and for 1 h after the treatment period (±70–71 h). Error bars of all free chlorine data points represent the standard error of triplicate readings at each time point (*n* = 3).

A dilution of commercial sodium hypochlorite containing 570 mg L^−1^ free chlorine was used to disrupt the biofilms. The effect of the perturbation was monitored on both biofilm metabolism and biomass (Fig. 6a). Although the exposure time was short (1 h, green region, Fig. 6a, b), the consequences were observed for several hours post event. The exposure caused a drastic and sustained reduction in both biofilm biomass and metabolic activity, with values for the two parameters reduced by 5% (biomass) and 25% (activity) during exposure (Fig. 6b). The substantial reduction in influent free chlorine concentration from 570 to 0.5 mg L^−1^ in the effluent during the first 30 min of exposure can be attributed to the oxidation of biofilm biomass. The effluent concentration of free chlorine increased to 22 mg L^−1^ (4% of initial free chlorine) at the end of the 1 h exposure and peaked at 30.0 mg L^−1^, 15 min post treatment. The retention time in the system is ~20 min, which accounts for the delay in increase and decrease of free chlorine due to plug-flow hydrodynamics in the system. The concentration of free chlorine returned to pre-exposure levels within 90 min of the start of treatment (below detection limit). The prolonged effect of the perturbation, beyond the treatment period, is evident with a 25% reduction in biomass and 50% decrease in metabolic activity. The reintroduction of growth medium facilitated biofilm recovery within hours of cessation of treatment (74–120 h, white region to the right of the green region, Fig. 6). A rapid recovery in biofilm metabolic activity is observed, coupled with a slightly delayed recovery of biofilm biomass. Interestingly, biofilm biomass recovered and established at higher levels than that observed pre-exposure (113% higher) whereas the CO_2_ production rate of the recovered biofilm never reached pre-exposure levels (10% lower) with a new steady state of 90 μmol h^−1^ CO_2_ established during the remaining ±50 h of monitoring. It is possible that cells killed by the treatment were trapped in the biofilm matrix by new cells growing in the outer region, with the dead cells still accounted for in the biomass measurement, while not contributing to overall metabolic activity.

## Discussion

Biofilm investigations in the last decade have vastly changed our understanding of these multicellular sessile communities. Advances have been facilitated by the development and refinement of tools and techniques to study microbial biofilms in vitro and in vivo^15,19,25,26^. Despite this, the vast majority of methodologies only provide a temporal/periodic and destructive view into biofilm development, structures, and survival^19,26,27^. The rapid expansion of IoT technologies into the biological field has allowed the development of cost-effective, open-sourced equipment in garages, kitchens, and laboratories once thought to be highly specialized and limited to industry (PCR, qPCR, microscopes etc.) (https://openpcr.org/ and https://www.chaibio.com/)^20,28,29^.

Spectrophotometric measurements continue to be a mainstay of microbiology and while its application to biofilms cultivated in batch systems such as microtiter plates has provided valuable insights, it is mostly limited to temporal and destructive measurements^25,26,30–32^. Traditionally, incident light at wavelengths in the range of 595–600 nm^33^ are used since these longer wavelengths allow for the accurate measurement of absorbance by microbial populations without the damage and interference at a molecular level associated with shorter wavelengths of light (UV-Violet)^33–35^.

The use of continuous absorbance measurements to study biofilms has been investigated previously^34,35^, with Bakke et al. demonstrating that biofilm optical density measurements were correlated to biofilm biomass, measured as total organic carbon^34^. However, these studies utilized complicated, proprietary and expensive hardware/software, in addition to an acknowledged system limitation relating to the matching of the wavelength of light source and detector^35^. The development and refinement of a small, cost-effective, on-line biofilm spectrophotometric system (BioSpec) reported here, has allowed for the nondestructive, high resolution, and real-time monitoring of changes in biofilm biomass in a continuous manner. Recognizing the complexity of biofilms, we view the term “biofilm biomass” to comprise cellular and noncellular fractions of biotic origin, as well as inorganic matter. The linear correlation between absorbance measured by the BioSpec for a range of undiluted, highly turbid standards, and sensitivity on par with that of a conventional spectrophotometer, provides a promising avenue for the study of these complex systems.

The validation of the BioSpec against a commercial spectrophotometer using planktonic biomass was the first step towards the proposed biofilm application. Initially, serial dilution and plating to determine the concentration of planktonic cells was utilized as an independent measure of biomass. This proved problematic due to aggregate formation commonly observed with *Pseudomonas spp*^36,37^. and therefore total protein was used instead to provide an additional means of biomass quantification^38,39^. The range of absorbance values for planktonic biomass grown on 3 g L^−1^ TSB proved to be in the linear range of the conventional spectrophotometer without the need for sample dilution prior to measurement. To evaluate detection capabilities at higher biomass concentration, 10 g L^−1^ TSB was used to obtain higher biomass density in batch cultures. The performance of the BioSpec was on par with the conventional spectrophotometric readings for a range of planktonic biomass concentrations, with good linear correlations between absorbance measurements for these systems, showing promise for the application of BioSpec to sessile microbial populations. Most spectrophotometers operate in a linear detection range of 0.2–2 absorbance units, which provides a narrow functional range and requires concentration/dilution of samples that fall outside of the linear detection limits^40^. The validation of the BioSpec with undiluted, highly turbid samples was critically important because biofilm biomass cannot be diluted in situ.

The dynamic nature of microbial biofilms requires continuous, high frequency monitoring to facilitate a greater understanding of biofilm response to changing environmental conditions. The BioSpec facilitates this type of monitoring, either as a stand-alone system, or in combination with metabolic activity detection using whole-biofilm CO_2_ production with the CEMS. Although total protein is recognized as an accurate measure of biofilm biomass, it requires destructive sampling, which renders it unsuitable for continuous monitoring of biofilms. The strong linear correlation between total protein and BioSpec measurements from sessile populations further strengthens the application of the system to accurately and nondestructively monitor changes in biofilm biomass in real time without the need for labor intensive sampling. The aim of the BioSpec system is not to quantify biofilm biomass by correlating it to biofilm thickness measurements for example, but rather to monitor relative changes in attached biomass. The quantification of biofilm biomass with optical density measurements would require significant optimization for each change in experimental conditions and/or microorganism(s). The use of relative measurements, rather than absolute quantification, may therefore be of greater value^34^.

Previous publications from our group demonstrated that the monitoring of biofilm metabolic activity with CEMS under continuous flow conditions in a nondestructive, real-time, and high-resolution manner, yields valuable insight into the interplay between biofilm proliferation and survival^15,39,41–43^. While these insights are valuable, CEMS cannot detect the biofilm biomass responsible for producing the detected CO_2_, and importantly do not reveal the efficiency of antibiotics or biocides/dispersants to remove protective biofilm biomass.

The importance of multiparameter monitoring is emphasized when examining the differential response of biofilm metabolism and biomass observed during changes in nutrient availability. *Pseudomonas aeruginosa* PAO1 biofilms initially cultivated on 3 g L^−1^ TSB reached a steady state with respect to metabolic activity notably earlier than that of biomass (Fig. 5). This may suggest that metabolism reached a maximal level for both sessile and planktonic cells under the prevailing nutritional supply, whilst continuing to support biomass accumulation (i.e., cell and EPS production). The subsequent increase in nutrient availability to 10 g L^−1^ TSB resulted in higher metabolic rates but did not translate into a large increase in biofilm biomass since the optimal biofilm structure (i.e., density and thickness) was likely already reached for the prevailing hydrodynamic flow conditions and associated forces. Reducing the nutrient concentration from 10 to 6 g L^−1^, and finally to 3 g L^−1^ TSB resulted in metabolism and biomass returning to the steady-state values achieved during initial biofilm establishment. This supports the suggestion that the limited increase in biomass following the shift from 3 to 10 g L^−1^ TSB can be explained by physical constraints on biomass related to hydrodynamic, and other factors such as nutrient/metabolite transport through the biofilm matrix that is mostly restricted to diffusion. These different metabolic and biomass responses, coupled to the lack of complete biomass erosion/detachment during metabolic dormancy observed during the carbon and nitrogen limitation period, strengthens the notion that biofilms are complex community structures^44^ that have evolved to rapidly react to environmental changes, and cautions against our customary dependence on batch systems to evaluate their role in the interplay between survival and proliferation.

Biofilms are responsible for substantial biofouling problems in various settings, ranging from food processing to healthcare facilities^2,5,9,27^. The use of chemical control measures to manage biofouling is a well-established field of research and business, with large multinational companies heavily invested in biocide/biodispersant development and manufacturing^45,46^. Production outputs, patient outcomes and overall human safety is dependent on effective control and management of biofilms, with correct formulation and dosing being critical to the success of control strategies. While biofilm control strategies are applied in many environments, it often proves to be either temporarily effective or entirely ineffective due to the recalcitrant nature of biofilms^47–49^.

The US EPA guidelines recommend a sodium hypochlorite concentration of 570 ppm (1:100 dilution) to effectively disinfect blood spills on hospital surfaces (https://www.cdc.gov/infectioncontrol/guidelines/disinfection/disinfection-methods/chemical.html)^50^, with contact times recommended by suppliers typically varying between 10 and 60 min. Similar variation exists for other antimicrobials and different environments, and the results presented here support numerous reports on the failure to treat biofilms with clinical and industrial relevance, despite following the guidelines on dosing concentration and exposure time. The rapid biofilm response and recovery measured after exposure in this study can be (at least partially) attributed to the inadequate removal of biofilm biomass from the system as shown in Fig. 6. It has been suggested that biofilm persistence has driven the search for improved or novel mitigation and control strategies without necessarily taking into account the complex and resilient nature of biofilms^25,47^. Such resilience was illustrated here by the rapid and interdependent response of biofilm structure and activity to environmental changes. The combination of BioSpec and CEMS facilitates the simultaneous measurement of two important independent biofilm metrics. When combined with other tools such as molecular markers and addition of different LED/sensor modules, this should be of value in future fundamental studies on biofilm form–function relationships, and further development may facilitate the use of the BioSpec as a tool to optimize biofilm control strategies.

Overall, biofilms represent a complex microbial form of existence and provide challenges to understanding, managing, and preventing/establishing their formation in various environments important to society. The need to progress from efforts aimed at delineating biofilm behavior by using a reductionist approach, as evident from biofilm studies using hydrodynamically static model systems, to a more representative mode is emphasized by the persistence of biofilm-linked food contamination and nosocomial infections, despite the implementation of best practice procedures^51–54^. The approach described here provides a simple, reliable and cost-effective tool for the study of biofilm biomass with the possibility of high-resolution monitoring in real-time without disturbing the biofilm’s structure. The open-sourced nature of the system benefits from the use of low cost, high quality components. The entire BioSpec system was constructed for <$200, compared to conventional spectrophotometers costing in excess of $5000^23,24^. The combination of BioSpec and CEMS facilitates the simultaneous measurement of two important biofilm metrics, which provides a basis for studies aimed at investigating biofilm form–function relationships, and with minor modification will facilitate transcriptomics and metabolomics at each stage of biofilm establishment/response to environmental changes. The application of the BioSpec, by itself or in combination with the CEMS system, should find relevance beyond fundamental research as it may provide valuable insight into the evaluation of existing disinfection procedures and development of novel strategies to manage microbial biofilms.

## Methods

### Bacterial strain and growth conditions

*Pseudomonas aeruginosa* PAO1 (South African National Health Laboratory Service, Gauteng, SA) was cultivated and maintained on 3 g L^−1^ TSB under aerobic conditions at 26 °C unless otherwise stated.

### Biofilm biomass monitoring system (BioSpec)

**Fig. 7:**
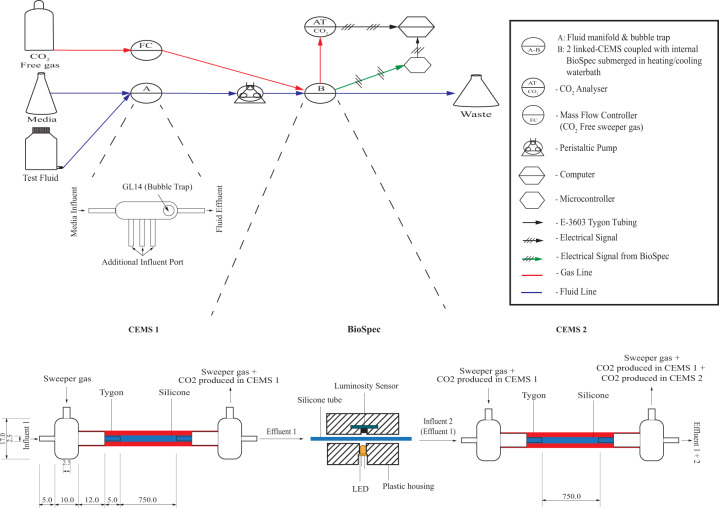
Schematic diagram of the CEMS-BioSpec system. Culture media and test fluids were introduced via a manifold (**a**) from the respective reservoirs with a peristaltic (**b**) to the respective systems contained in a heating/cooling water bath (26 °C). The CO_2_-Free sweeper gas was introduced into the annular space of CEMS (Expanded B, red shaded region) via a gas mass flow controller, allowing for the collection of biofilm-evolved CO_2_ and analyzed by an infrared CO_2_ analyzer. Biofilm biomass was measured between the two CEMS units (expanded B) via the internal silicon tube (containing biofilm biomass) being passed through a cavity with a LED illuminating the tube from one side and a digital light sensor on the transverse side measuring the amount of illumination absorbed by biomass in the tube. All dimensions are to the nearest mm.

A biofilm biomass monitoring systems (BioSpec) was developed through the coupling of photometric sensors to a computer via microcontrollers (Fig. 7).

Hardware of the BioSpec system consists of two modular components, the module that measures biomass and the datalogging module. The biomass module consists of an advanced digital light sensor TSL2561 (Adafruit industries, NY, USA) placed perpendicular with a 595 nm LED (Cree C503B-AAN) (Supplementary Information Fig. 1) with a narrow spectral bandwidth (±550 to 650 nm) and peak wavelength around 595 nm (591 to 596 nm) (RS Components SA, Midrand, SA) with the sensor and LED being separated by silicone tubing (I.D. 1.6 mm and O.D. 2.5 mm). The above components were all housed in a custom, 3-D printed ABS plastic housing designed and printed in house (Fig. 7 and Supplementary Information Fig. 2).

The datalogging module is comprised of an Arduino™ Mega ADK microcontroller (https://www.arduino.cc/) interfaced through a custom printed circuit board (Supplementary Information Fig. 3) that allows for the multiplexing of multiple light/luminosity sensors, connected to a personal computer for data retention and processing.

Customization of the open source codes and compilations were done in Arduino’s Integrated Development Environment.

The data logged by the light sensor was provided in the illuminance SI units of lux. The lux value was converted to the unitless value of absorbance (conventional spectrophotometric value) using the following equation (Eq. 1) (https://www.edinst.com/blog/the-beer-lambert-law/)^30^.

*A* = log_10_ *I*_0_/*I* (Eq. 1) where

*A* = Amount of absorbed light

*I*_0_ = Incident light*

*I* = Transmitted light

*Measured at the start of each experiment using clean silicone tubing filled with sterile distilled water, to account for any potential variation in light intensity, sensor sensitivity and/or variation in silicone tubing that may occur between experiments.

### Validation of BioSpec

For turbidity assays, a range of 0.5–10 McFarland standards were freshly prepared as previously described (https://www.dalynn.com/dyn/ck_assets/files/tech/TM59.pdf). Each McFarland standard was thoroughly mixed, and 3 mL injected into the silicone tubing of the BioSpec. Changes in light intensity (lux at 595 nm) were monitored at 10 s intervals for 30 s (triplicate readings). The McFarland standards were sequentially introduced in increasing order of turbidity. The silicone tube was rinsed twice with distilled water between injections. Absorbance readings (OD at 595 nm) of the McFarland standards were also measured using disposable 2 mL cuvettes and a SpectroQuant^®^ spectrophotometer (Merck Millipore, Gauteng, SA). Readings were in triplicate.

### Planktonic and sessile growth determined with BioSpec

Planktonic growth was determined in duplicate 250 mL flasks containing 100 mL of either 3 or 10 g L^−1^ sterile TSB were inoculated with *P. aeruginosa* PAO1 pre-cultured to an optical density (OD_595nm_ = 0.1) and incubated aerobically on an orbital shaker at 150 rpm at 26 °C for 24–48 h. Samples were aseptically removed from the flasks at hourly intervals during the first 12 h of incubation, followed by less frequent sampling. Each sample was analyzed for changes in turbidity using the BioSpec and a spectrophotometer, respectively, as described above.

Conventional cell counts were initially used as an independent measure of biomass accumulation but proved to be inaccurate due to the aggregative phenotype of *Pseudomonas* spp^37^. Total protein concentration was thus used as a proxy for biomass accumulation as previously described^39^. Briefly, 6 mL of the culture was removed from each flask at the respective time intervals, the biomass harvested (10,000 × *g*, 5 min), resuspended in 0.3 mL 0.1 N NaOH solution and incubated at 70 °C for 1 h. The total protein content of each sample was determined using a Pierce BCA protein assay kit (Thermo Scientific, Rockford, IL, USA), according to the manufacturer’s instructions. All sampling points represent the mean of biological duplicates and technical triplicates (*n* = 6).

Sessile growth dynamics were studied under continuous flow conditions within the CEMS^15^ linked to the BioSpec, allowing for the simultaneous real time, nondestructive monitoring of biofilm metabolism, and biomass respectively (Fig. 7). Two CEMS systems were constructed as previously described^15,55^, with slight modification. In essence, the CEMS system is based on the high CO_2_ permeability of silicone (permeability coefficient of 20 132) vs. near-impermeable tygon (permeability coefficient of 270); using silicone tubing as a continuous-flow bioreactor encased in a tygon tube through which a sweeper gas continuously carries the microbially-produced CO_2_ to a CO_2_ analyzer. The BioSpec was positioned between two 75 cm-long systems (half the length of the original 1.5 m-long silicone tubing between inlet and outlet) (Fig. 7). The silicone tube was disinfected with 3.5% (vol/vol) sodium hypochlorite for 2 h and then rinsed overnight with sterile distilled water. The silicone tubing was filled with sterile 10 g L^−1^ TSB, using a Watson Marlow 205S peristaltic pump. The system was inoculated by ceasing medium flow to the system, followed by injecting 1.0 mL of *P. aeruginosa* PAO1 standardized to OD_595nm_ = 0.1. The flow was restored after 30 min post inoculation at a flow rate of 12.5 mL h^−1^ and biofilms were grown to metabolic and biomass steady state (stabilization of values) at 26 °C.

In a separate experiment, eight pairs of 1.5 m-long silicone tubes (16 tubes with 1.6 mm I.D. and 2.5 mm O.D.; the same dimensions as the tubes used in the CEMS and BioSpec) were used for cultivation of *P. aeruginosa* PAO1 biofilms to determine total biofilm protein as an independent measurement of biofilm biomass. The tubing pairs were inoculated with *P. aeruginosa* PAO1 (OD_595nm_ = 0.1) and cultivated at 26 °C. One pair of the silicone tubes (1 of the 8 sets) were sacrificed at a time for total biofilm protein extraction. The timing of extraction was determined using the real-time monitoring of both biofilm biomass (BioSpec) and metabolic activity (CEMS) to allow for representative sampling during the establishment (lag), development (log) and steady-state (stationary) phases of biofilm growth. Total biofilm protein values represent the average of duplicate biofilms and triplicate technical repeats (*n* = 6).

### Simultaneous monitoring of the effects of perturbations on biofilm biomass and metabolic activity

The response of biofilms to alternating nutrient concentrations were monitored in the combined CEMS-BioSpec system. Sterile glass manifolds were installed upstream of the peristaltic pump to facilitate bubble-free, aseptic introduction of different nutrient concentrations to the biofilms. Biofilms were cultivated on 3 g L^−1^ TSB to steady state, before the sequential introduction of 10 g L^−1^, 0 g L^−1^ (no added carbon or nitrogen, only buffer components of TSB), 6 g L^−1^ and finally 3 g L^−1^ TSB. Biofilms were allowed to reach a new steady state (with respect to biomass and metabolic activity), prior to the introduction of the following nutrient concentration.

Sodium hypochlorite was used to initiate a chemical perturbation of the biofilm. Sodium hypochlorite was applied at 570 ppm in dH_2_O (1:100 dilution) as recommended by the Centers for Disease Control and Prevention (CDC), Environmental Protection Agency (EPA) and World Health Organization (WHO) for disinfection^50,56,57^. Dosing was conducted using the sterile glass fluid manifold. The dosing period was limited to 60 min as this corresponds to the longest disinfection period recommended in the CDC, EPA and WHO guidelines for disinfection using a 1:100 dilution)^50^^,^^56,57^. Free chlorine concentrations were determined using the SpectroQuant^®^ chlorine test kit (Merck Millipore, 100599 chlorine kit) as per the manufacturer’s instructions. The free chlorine concentration of the diluted sodium hypochlorite solution was determined prior to introducing it into the glass manifold upstream of the CEMS-BioSpec system, and thereafter in the effluent from the system, during the dosing period and for 1 h after the exposure period. Briefly, samples were combined with the contents of the kit and concentrations were determined spectrophotometrically (SpectroQuant^®^). A recovery period was initiated after the 60 min dosing period by the reintroduction of sterile 3 g L^−1^ TSB to monitor changes in biofilm metabolic activity and biomass. All free chlorine data points represent the mean of triplicate readings at each time point (*n* = 3).

### Statistical analyses

Linear regression analysis and Pearson correlation and coefficient determination (*r* and *R*^2^, *p* < 0.05) for the turbidity assays were conducted utilizing the IBM SPSS 22 software package. Where appropriate, all vertical error bars represent standard deviation and sample sizes are indicated in parentheses.

### Reporting summary

Further information on research design is available in the Nature Research Reporting Summary linked to this article.

## Supplementary information

Supplementary Information

Reporting Summary

## Data Availability

The authors declare that all data supporting the findings of this study are available within the paper and its Supplementary Information File.

## Code availability

The authors have made the code utilized within the paper available under the GNU General Public License v3.0 agreement on GitHub under the following link https://github.com/KBKlopper/BioSpec.git.
