# Supplementary material for: Biofilm dynamics: linking in situ biofilm biomass and metabolic activity measurements in real-time under continuous flow conditions
Source: NPJ Biofilms Microbiomes. 2020 Oct 21;6:42. doi: 10.1038/s41522-020-00153-9 (PMC7578832; doi:10.1038/s41522-020-00153-9)
Supplement: Supplementary file 1 — Supplementary Information [file 41522_2020_153_MOESM1_ESM.pdf]

Supplementary Information: BioSpec and CEMS: Combining *in-situ* biofilm biomass and metabolic activity measurements in real time under continuous flow conditions.

Kyle B. Klopper[1], Riaan N. de Witt[1], Elanna Bester[1], Leon M. T. Dicks, Gideon M. Wolfaardt[1,2*]

[1]Department of Microbiology, Stellenbosch University, Stellenbosch, South Africa

[2]Department of Chemistry and Biology, Ryerson University, Toronto, ON, Canada

[*] GMW@sun.ac.za

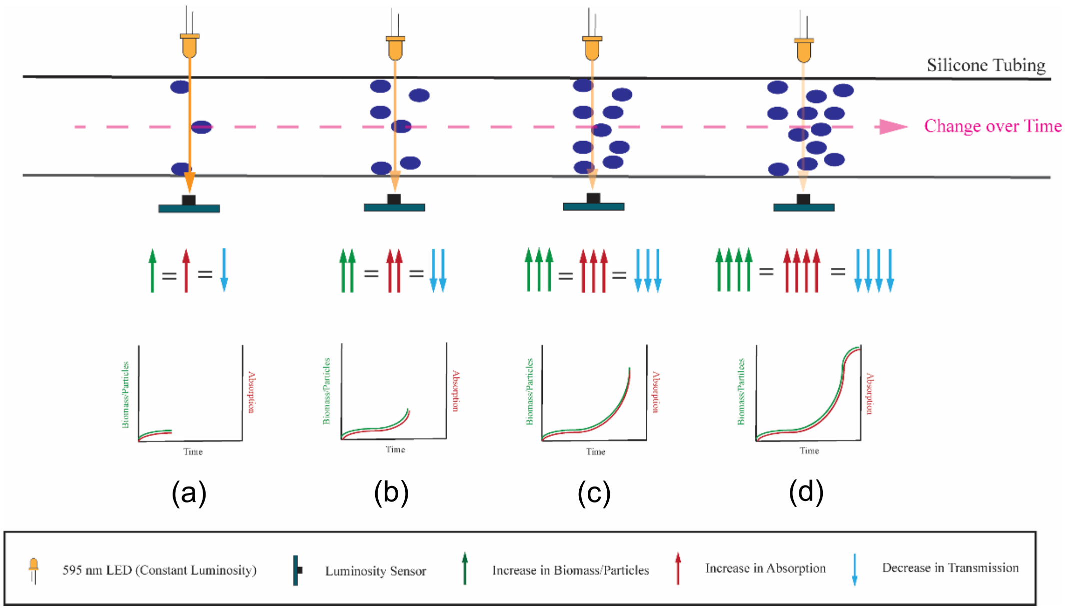

Supplementary Figure 1. Graphical representation of the principle utilized by the BioSpec system to monitor biofilm biomass changes. (a) During the initial biofilm establishment (lag) phase, a minimal decrease in transmission is detected (i.e. a slight increase in absorption) **(b)** Start of the log phase when substantial and rapid production of biomass attached to the inner surfaces of the silicone tubing result in a notable decrease in transmission (i.e. increase in absorption). **(c)** The tail-end of the log phase where the rate of biofilm biomass production slows and is detected as a corresponding decrease in light transmission through the tubing. **(d)** The stationary phase where biofilm biomass has reached steady-state and results in a plateau in absorption values detected by the system.

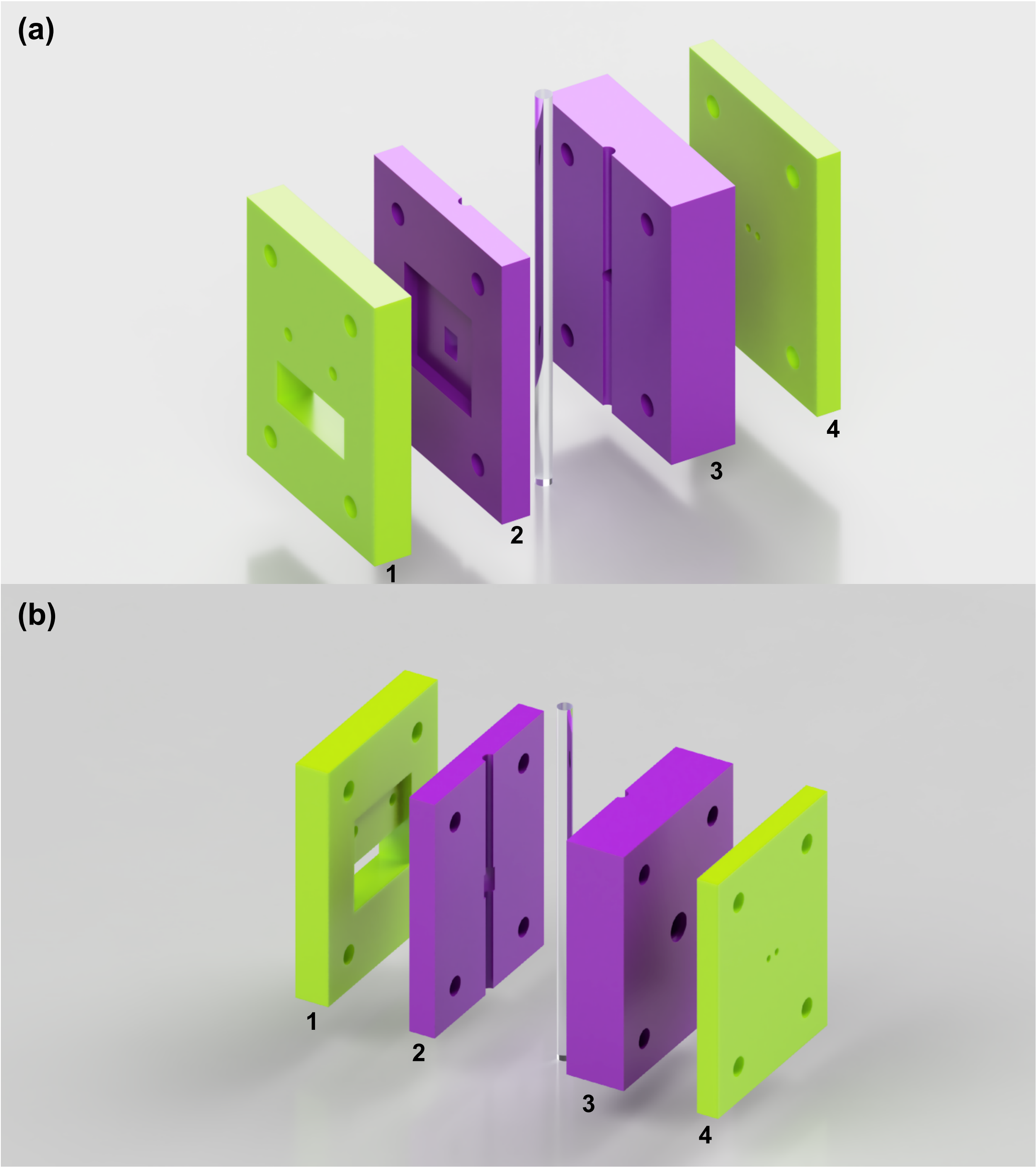

Supplementary Figure 2. The BioSpec sensor housing and assembly

Graphical rendering of the 4 parts printed and assembled to form the BioSpec sensor units. **(a)** The right-hand orthographic view of BioSpec sensor unit illustrating sensor placement in parts 1 & 2. The luminosity sensor is mounted with M2.5 fasteners and bolts to part 1. Part 2 contains

an opening to allow light transmitted through the silicone tubing to reach the luminosity sensor mounted in part 1. The silicone tubing, represented by the clear cylinder between parts 2 and 3 is secured in place by the vertical groove printed in parts 2 and 3. **(b)** The left-hand orthographic view of BioSpec sensor unit illustrating LED placement (parts 3 & 4). The 595 nm LED is press fitted into part 3 and secured in place by part 4. The LED contacts pass through openings in part 4. The entire sensor unit is assembled with 4 X M4 fasteners and nuts to form a single module.

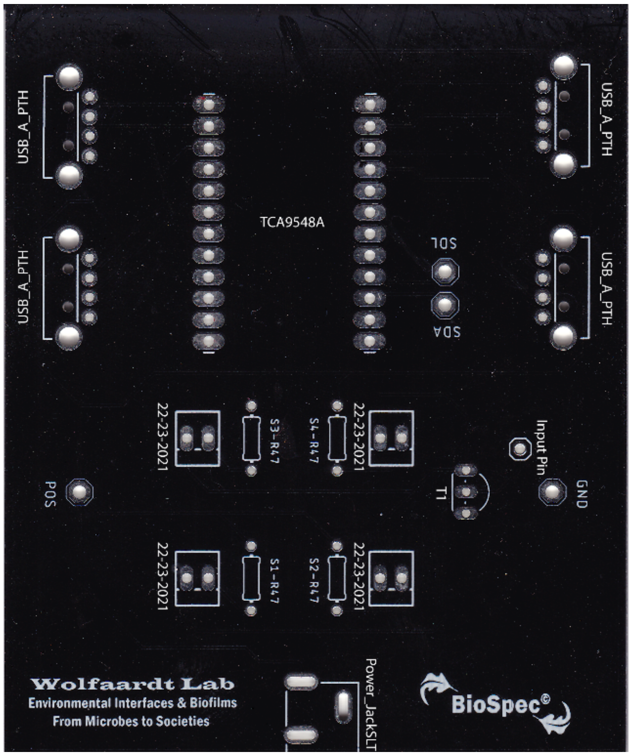

Supplementary Figure 3. BioSpec PCB and component layout. Component placement on PCB is indicated on the board in white text according to the component codes contained within the BOM. The PCBs are manufactured without components and components are purchased separately and soldered to the board in the corresponding location.
