# Supplementary material for: Biofilm dynamics: linking in situ biofilm biomass and metabolic activity measurements in real-time under continuous flow conditions
Source: NPJ Biofilms Microbiomes. 2020 Oct 21;6:42. doi: 10.1038/s41522-020-00153-9 (PMC7578832; doi:10.1038/s41522-020-00153-9)
Supplement: Supplementary file 2 — Reporting Summary [file 41522_2020_153_MOESM2_ESM.pdf]

nature research

Corresponding author(s): GM Wolfaardt

Last updated by author(s): Aug 29, 2020

Reporting Summary

Nature Research wishes to improve the reproducibility of the work that we publish. This form provides structure for consistency and transparency in reporting. For further information on Nature Research policies, see our Editorial Policies and the Editorial Policy Checklist.

Statistics

For all statistical analyses, confirm that the following items are present in the figure legend, table legend, main text, or Methods section.

n/a	Confirmed	
☐	☒	The exact sample size (*n*) for each experimental group/condition, given as a discrete number and unit of measurement
☐	☒	A statement on whether measurements were taken from distinct samples or whether the same sample was measured repeatedly
☐	☒	The statistical test(s) used AND whether they are one- or two-sided<br>*Only common tests should be described solely by name; describe more complex techniques in the Methods section.*
☐	☒	A description of all covariates tested
☐	☒	A description of any assumptions or corrections, such as tests of normality and adjustment for multiple comparisons
☐	☒	A full description of the statistical parameters including central tendency (e.g. means) or other basic estimates (e.g. regression coefficient) AND variation (e.g. standard deviation) or associated estimates of uncertainty (e.g. confidence intervals)
☒	☐	For null hypothesis testing, the test statistic (e.g. *F*, *t*, *r*) with confidence intervals, effect sizes, degrees of freedom and *P* value noted<br>*Give P values as exact values whenever suitable.*
☒	☐	For Bayesian analysis, information on the choice of priors and Markov chain Monte Carlo settings
☒	☐	For hierarchical and complex designs, identification of the appropriate level for tests and full reporting of outcomes
☐	☒	Estimates of effect sizes (e.g. Cohen's *d*, Pearson's *r*), indicating how they were calculated

Our web collection on statistics for biologists contains articles on many of the points above.

Software and code

Policy information about availability of computer code

Data collection	Customization of the open source codes and compilations were done in Arduino's Integrated Development Environment. Lux value was converted to the unitless value of absorbance, following equation obtained at https://www.edinst.com/blog/the-beer-lambert-law/
Data analysis	Linear regression analysis, Pearson correlation & coefficient determination (r & R2, p < 0.05) for the turbidity assays were conducted utilizing the IBM SPSS 22 software package

For manuscripts utilizing custom algorithms or software that are central to the research but not yet described in published literature, software must be made available to editors and reviewers. We strongly encourage code deposition in a community repository (e.g. GitHub). See the Nature Research guidelines for submitting code & software for further information.

Data

Policy information about availability of data

All manuscripts must include a data availability statement. This statement should provide the following information, where applicable:

- Accession codes, unique identifiers, or web links for publicly available datasets
- A list of figures that have associated raw data
- A description of any restrictions on data availability

The authors declare that all data supporting the findings of this study are available within the paper and its supplementary information files. The authors have made the code utilized within the paper available under the GNU General Public License v3.0 agreement on GitHub under the following link https://github.com/KBKlopper/BioSpec.git.

Field-specific reporting

Please select the one below that is the best fit for your research. If you are not sure, read the appropriate sections before making your selection.

☒ Life sciences ☐ Behavioural & social sciences ☐ Ecological, evolutionary & environmental sciences

For a reference copy of the document with all sections, see nature.com/documents/nr-reporting-summary-flat.pdf

Life sciences study design

All studies must disclose on these points even when the disclosure is negative.

Sample size	Turbidity assay were performed in triplicate. All data for planktonic and sessile growth assessments represent the mean of biological duplicates and technical triplicates (n = 6)
Data exclusions	There are no data exclusions
Replication	All attemts to replicate were successful
Randomization	The method described here is performed on-line / real-time, thus no random sampling.
Blinding	The method described here is performed on-line / real-time, thus no blinding procedures apply.

Reporting for specific materials, systems and methods

We require information from authors about some types of materials, experimental systems and methods used in many studies. Here, indicate whether each material, system or method listed is relevant to your study. If you are not sure if a list item applies to your research, read the appropriate section before selecting a response.

Materials & experimental systems

n/a	Involved in the study	
☒	☐	Antibodies
☒	☐	Eukaryotic cell lines
☒	☐	Palaeontology and archaeology
☐	☒	Animals and other organisms
☒	☐	Human research participants
☒	☐	Clinical data
☒	☐	Dual use research of concern

Methods

n/a	Involved in the study	
☒	☐	ChIP-seq
☒	☐	Flow cytometry
☒	☐	MRI-based neuroimaging

Animals and other organisms

Policy information about studies involving animals; ARRIVE guidelines recommended for reporting animal research

Laboratory animals	Pseudomonas aeruginosa PA01
Wild animals	No animals were used
Field-collected samples	A lab strain was used
Ethics oversight	No ethical approval required

Note that full information on the approval of the study protocol must also be provided in the manuscript.
